# Human cardiovascular organoids: Biomedical applications and ethical challenges

**DOI:** 10.1016/j.ahjo.2026.100882

**Published:** 2026-09-07

**Authors:** Koushik Sen, Suravi Majumder, Dragana Stanisic, Avisek Majumder

**Affiliations:** a Department of Zoology, Jhargram Raj College, 721507, Jhargram, West Bengal, India; b Department of Pharmacological and Pharmaceutical Sciences, University of Houston, Houston, TX, 77004, USA; c Department of Dentistry, Faculty of Medical Sciences, University of Kragujevac, 34000, Kragujevac, Serbia; d Department of Medicine, University of California, San Francisco, CA, 94158, USA

**Keywords:** Human pluripotent stem cells, Cardiac organoids, Engineered cardiac tissues, Disease modelling, Drug screening, Regenerative medicine, Precision medicine, Cardiac bioengineering

## Abstract

Cardiovascular disease (CVD) remains the leading cause of global mortality, yet traditional in vitro and animal models often fail to recapitulate the integrated structural, cellular, and functional complexity of the human heart. Recent advances in human pluripotent stem cell technology, developmental bioengineering, and microphysiological systems have enabled the development of diverse three-dimensional cardiac tissue models, including self-organizing cardiovascular organoids and engineered cardiac platforms, three-dimensional constructs that partially recapitulate key myocardial features. These platforms facilitate mechanistic and disease-relevant modelling of congenital and acquired cardiac disorders, allow interrogation of arrhythmogenic mechanisms, and reproduce pathophysiological processes such as ischemia–reperfusion injury, fibrotic remodelling, and inflammatory cardiomyopathy with increasing physiological relevance. In translational contexts, these models are emerging as valuable tools in drug discovery pipelines, enabling high-throughput cardiotoxicity assessment and supporting regenerative medicine strategies, thereby bridging mechanistic research with preclinical validation. Integration with cutting-edge modalities—such as single-cell and spatial multi-omics, CRISPR-based genome editing, synthetic biology circuits, and artificial intelligence-driven phenomics further enhances their maturation and predictive accuracy, although their clinical applicability remains under active investigation. Despite these advances, challenges including batch-to-batch variability, limited vascularization, metabolic immaturity, and incomplete immune system incorporation continue to constrain physiological fidelity. Additionally, ethical and regulatory considerations spanning donor consent, genetic data privacy, biobanking governance, and model-specific translational and societal implications remain critical for responsible innovation. This review presents a comprehensive synthesis of current cardiovascular organoid research, highlights emerging biomedical applications, and provides a critical perspective on the ethical frameworks essential for advancing the field responsibly.

## 1 Introduction

Cardiovascular disease (CVD) remains the leading cause of morbidity and mortality worldwide and continues to impose a major global health burden. Current estimates indicate that more than 523 million individuals are living with cardiovascular disorders, while approximately 17 million deaths annually are attributed to cardiovascular causes, accounting for nearly one-third of global mortality [1], [2], [3], [4]. Despite substantial advances in diagnostic imaging, surgical interventions, pharmacotherapy, and cardiovascular risk management, the development of effective disease-modifying therapies has slowed in several areas of cardiology. One important limitation is the lack of experimentally relevant human cardiac models capable of adequately reproducing the structural, multicellular, and electrophysiological complexity of native cardiac tissue [5], [6]. Traditionally, cardiovascular research has relied heavily on two-dimensional (2D) cell culture systems and animal models [7], [8], [9]. Although 2D cardiomyocyte cultures remain useful for basic electrophysiological and pharmacological studies, they do not fully reproduce the three-dimensional architecture, biomechanical forces, extracellular matrix interactions, and multicellular communication observed in human myocardium. Similarly, animal models have provided important insights into cardiovascular physiology and disease mechanisms but remain limited by interspecies differences in cardiac electrophysiology, metabolism, immune responses, and drug sensitivity [7], [10], [11]. These limitations contribute to discrepancies between preclinical findings and clinical outcomes, thereby restricting translational predictability in cardiovascular research.

Advances in stem cell biology and three-dimensional (3D) tissue culture technologies have enabled the development of more physiologically relevant in vitro cardiac systems. Following the pioneering introduction of induced pluripotent stem cell (iPSC) technology by Takahashi and Yamanaka in 2006, patient-specific cardiomyocytes and multicellular cardiac constructs became increasingly accessible for experimental investigation [12], [13]. Building upon these advances, researchers have developed cardiac organoids and engineered cardiac tissue platforms capable of reproducing selected developmental, structural, and functional features of human cardiac tissue. These systems incorporate varying combinations of cardiomyocytes, endothelial cells, fibroblasts, stromal populations, extracellular matrices, and bioengineering approaches to model aspects of cardiac morphogenesis, disease progression, and pharmacological response under controlled experimental conditions.

Cardiovascular organoids and engineered cardiac tissues have emerged as versatile human-specific platforms for investigating inherited cardiomyopathies, ischemic heart disease, cardiac arrhythmias, fibrotic remodelling, drug-induced cardiotoxicity, and regenerative cardiovascular therapies [14], [15]. Patient-specific iPSC-derived systems additionally support investigation of genotype-specific disease mechanisms and individualized therapeutic responses relevant to precision medicine applications [16], [17]. Parallel advances in bioengineering, including organ-on-chip systems, scaffold-based tissue engineering, vascularized culture platforms, and computational modelling, have further improved the structural and functional complexity of these experimental systems [18], [19], [20].

Despite substantial progress, cardiovascular organoids remain limited by developmental immaturity, incomplete vascularization, batch-to-batch variability, and restricted long-term functional stability, while challenges related to standardization, scalability, and clinical translation remain unresolved. Nevertheless, these platforms have significantly advanced human-specific modelling of cardiovascular development and disease. This review summarizes recent progress in cardiovascular organoids, highlighting advances in bioengineering, disease modelling, drug discovery, precision medicine, and regenerative research, while critically discussing the biological and translational challenges that must be addressed to facilitate their responsible clinical application.

## 2 Methodology

This review was conducted using a structured narrative and integrative literature-review approach to evaluate recent advances in cardiovascular organoid and engineered cardiac tissue research. Literature was comprehensively searched using major scientific databases, including PubMed, Scopus, Web of Science, and Google Scholar. Search terms included combinations of “cardiac organoid,” “cardiovascular organoid,” “heart organoid,” “engineered cardiac tissue,” “heart-on-chip,” “disease modelling,” “drug screening,” “cardiotoxicity,” “gene therapy,” and “regenerative cardiology.”

Recent publications from 2018 onwards were prioritized to capture advances in stem cell-derived cardiovascular modelling, while earlier landmark studies were included where necessary to provide historical and conceptual context. Relevant articles were initially identified through title and abstract screening, followed by full-text evaluation for their relevance to the scope of this review. Preference was given to peer-reviewed studies involving human pluripotent stem cell-derived cardiovascular organoids, engineered cardiac tissues, vascularized constructs, and translational bioengineering platforms. Studies focused exclusively on non-cardiac organoids, non-human experimental systems lacking translational relevance, or reports with insufficient methodological detail were not prioritized for detailed discussion. Selected articles were critically evaluated based on scientific quality, methodological rigor, reproducibility, translational relevance, multicellular complexity, and integration of functional validation approaches, including electrophysiological assessment, vascularization, transcriptomic analyses, and pharmacological testing. Particular emphasis was placed on studies addressing disease modelling, cardiotoxicity evaluation, regenerative applications, and emerging bioengineering strategies such as microfluidic platforms, bioprinting, and computational modelling.

The findings synthesized in this review provide a balanced overview of current advances, unresolved challenges, and future translational perspectives in cardiovascular organoid research. As a structured narrative review, this article integrates representative landmark studies, recent technological developments, and emerging translational advances to provide a comprehensive and balanced perspective on the rapidly evolving field of cardiovascular organoid biology.

## 3 Approaches and platforms for cardiac tissue modelling

### 3.1 Conceptual framework and classification of cardiac models

**Fig. 1:**
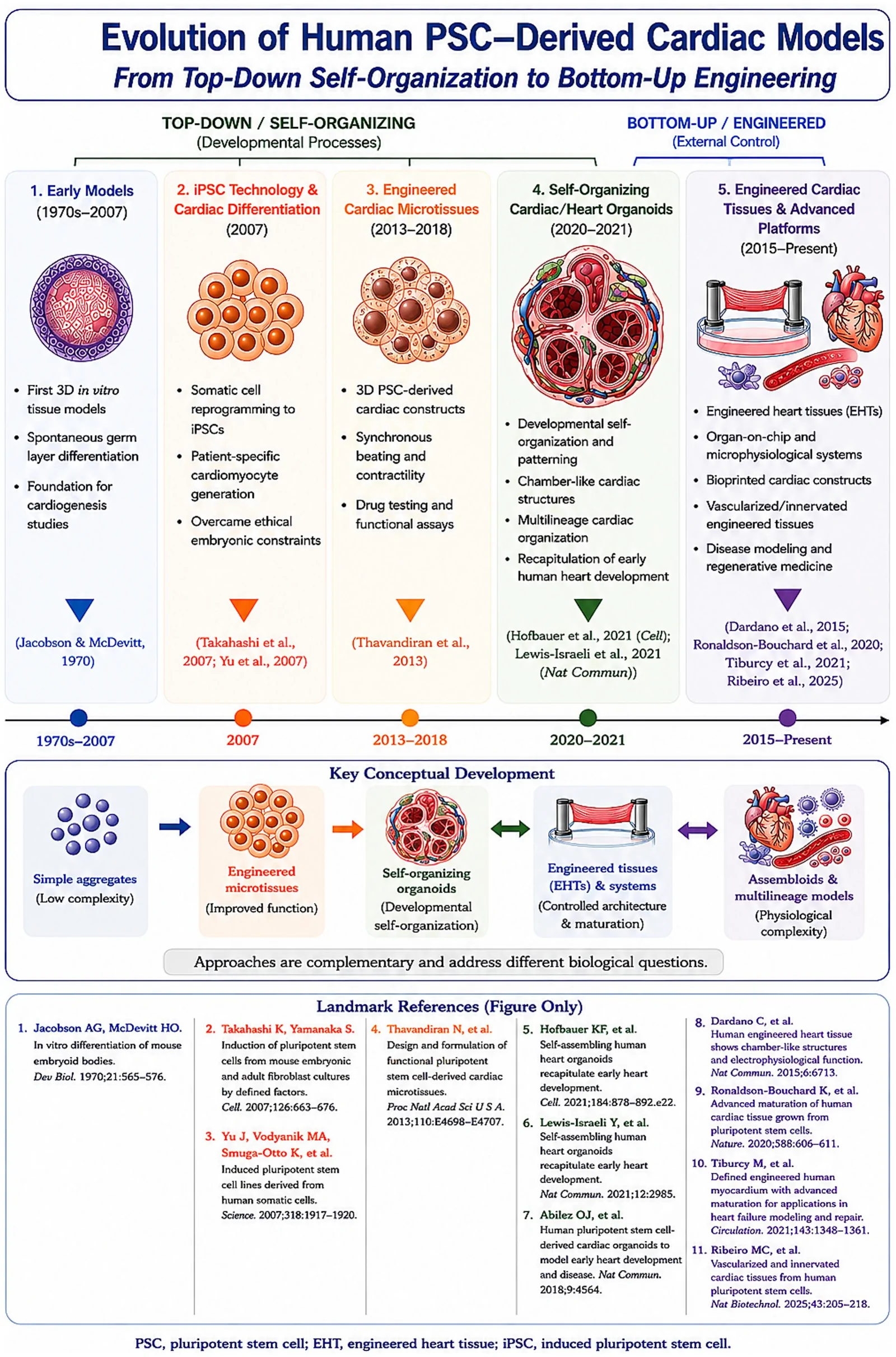
Evolution of human PSC-derived cardiac model platforms. Schematic overview of the evolution of human pluripotent stem cell (PSC)-derived cardiac models from early three-dimensional aggregates and cardiac microtissues to self-organizing cardiac organoids, engineered heart tissues and advanced multilineage and vascularized models. The framework distinguishes top-down, self-organizing approaches from bottom-up or externally engineered approaches, while recognizing that these developments have occurred in parallel and remain complementary. Self-organizing cardiac/heart organoids are highlighted as a distinct developmental approach characterized by intrinsic patterning and chamber-like organization. Later multilineage and vascularized systems further increase cellular and physiological complexity.

The rapid development of 3D-cardiac model systems has expanded the capacity to investigate human cardiac development, disease mechanisms, and therapeutic responses. However, this progress has also led to terminological inconsistency, as heterogeneous platforms are frequently grouped under the term “cardiac organoids” despite fundamental differences in their biological origin and degree of engineering control. This lack of distinction may result in misinterpretation of experimental findings and overestimation of physiological relevance. Accordingly, a clear conceptual framework is required to differentiate self-organizing, developmentally driven systems from engineering-based cardiac models (Fig. 1).

Self-organizing cardiac organoids are three-dimensional structures derived from pluripotent stem cells that undergo intrinsic self-organization and developmental patterning under defined culture conditions without imposed spatial pre-patterning [21], [22]. These models partially recapitulate key features of early human cardiogenesis through modulation of conserved signalling pathways, including Wnt, BMP, FGF, and retinoic acid [22], [23].

As a result, they form multicellular assemblies comprising cardiomyocytes, endothelial cells, and stromal populations, often exhibiting chamber-like organization, spontaneous contractility, and fetal-like electrophysiological properties [24], [25]. Their structural and functional organization arises predominantly from endogenous developmental programs, distinguishing them from engineered systems in which architecture is externally imposed [26], [27], [28].

Landmark studies introducing self-organizing cardiac organoids, including cardioids [22] and self-assembling human heart organoids [24], established these systems as developmentally relevant but immature platforms for studying early human cardiogenesis, morphogenesis, and congenital heart disease rather than adult myocardial physiology [22], [29], [30]. Recent advances have further expanded self-organizing cardiac organoids into increasingly complex multilineage developmental systems. Blood-generating heart-forming organoids (BG-HFOs) developed by Dardano et al. integrate cardiac, vascular, endothelial, and hematopoietic lineages within a single self-organizing platform, thereby recapitulating coordinated embryonic heart and hematopoietic co-development. These next-generation organoids extend the developmental complexity of conventional cardiac organoids and provide new opportunities for investigating early human cardiogenesis, hematopoietic specification, and multicellular developmental interactions [31].

Cardiac assembloids represent a complementary class of multicellular models in which separately specified or preformed cardiac and non-cardiac components are combined to investigate defined multicellular or inter-tissue interactions. These may include combinations of cardiac organoids or cardiac tissues with immune, neural crest, vascular, or other relevant cell populations. Unlike conventional self-organizing cardiac organoids, in which tissue organization arises predominantly from intrinsic developmental programs, assembloids are intentionally assembled from defined components, although individual components may retain self-organizing properties [32].

In contrast, engineered cardiac tissues (EHTs) are generated through the externally guided assembly of cardiac cells within predefined scaffolds, moulds, or mechanical supports [33], [34]. These systems prioritize tissue alignment, force generation, and reproducibility by imposing geometric, mechanical, and electrical constraints that guide cardiomyocyte organization and maturation. While EHTs enable quantitative assessment of contractility, electrophysiology, and pharmacological responses, they do not reproduce developmental self-organization or chamber morphogenesis. Instead, their structure and function are largely determined by engineering inputs, and they are best regarded as engineered myocardium rather than organoids [35], [36], [37].

Bioprinted cardiac constructs represent a distinct engineering-driven class in which cells and extracellular matrix components are spatially organized using additive manufacturing approaches. Bioinks composed of natural or synthetic polymers allow programmable control over tissue geometry, cellular distribution, and, in some cases, vascular architecture. In these systems, structural fidelity is primarily dictated by printing design rather than intrinsic morphogenetic processes. Although bioprinted tissues remain developmentally immature, they offer advantages for generating anatomically defined constructs and vascularized patches for regenerative applications and in vitro disease modelling [38], [39], [40], [41].

Microfluidic cardiac organ-on-chip systems are engineered platforms that culture cardiac cells or microtissues within microfabricated environments under controlled biochemical and biomechanical conditions. By integrating fluid flow, shear stress, cyclic strain, and electrical stimulation, these systems enable precise modelling of physiological parameters and functional readouts, including electrophysiology and contractility. Unlike organoids, organ-on-chip platforms do not aim to reproduce developmental morphogenesis but instead provide controlled environments in which biological components are studied within predefined engineering frameworks [42], [43].

These platforms represent complementary rather than interchangeable approaches to modelling human cardiovascular biology. Their distinct biological principles determine their respective strengths, with cardiac organoids recapitulating developmental self-organization and engineered systems providing greater control over tissue architecture and functional assessment. Recognizing these conceptual distinctions is essential for selecting appropriate experimental models and accurately interpreting their biological and translational relevance.

### 3.2 Self-organizing cardiac organoids

Human cardiac organoids are characterized by their intrinsic capacity for self-assembly and developmental self-organization. Under defined biochemical and culture conditions, pluripotent stem cell (PSC)-derived populations spontaneously generate three-dimensional multicellular structures that partially recapitulate selected features of early human cardiogenesis [29], [44], [45]. These developmental processes are orchestrated by conserved signalling pathways, including Wnt, BMP, FGF, Notch, and retinoic acid (RA), which coordinate lineage specification, tissue patterning, and spatial organization during cardiac development [46], [47], [48], [49], [50]. Consequently, cardiac organoids reproduce aspects of embryonic heart development, including chamber-like organization, spontaneous contractility, and the emergence of epicardial- and endothelial-like populations under fetal-like metabolic conditions [30], [51], [52].

Transcriptomic and single-cell analyses have demonstrated that cardiac organoids share molecular characteristics with early fetal heart tissue, including expression of key cardiac transcriptional and structural markers such as NKX2-5, GATA4, MYH7, and TNNT2 [53], [54], [55], [56]. Functional modulation of RA and Wnt signalling further supports atrioventricular specification and cardiomyocyte differentiation [23]. In addition, exposure to ondansetron disrupts developmental patterning and chamber or trabecular organization in cardiac organoids, consistent with drug-associated congenital cardiotoxicity observed in vivo [57], [58], [59], [60].

An important feature of this self-organizing process is the coordinated interaction among cardiomyocytes, endothelial cells, fibroblasts, and other stromal cell populations. Through endothelial–stromal and epicardial–myocardial crosstalk, these multicellular systems can develop primitive microvascular-like networks and extracellular matrix-rich microenvironments that help maintain tissue organization and support partial structural and functional maturation [61], [62], [63], [64], [65]. Such multicellular organization also enables modelling of inflammatory and fibrotic responses, thereby extending organoid applications beyond developmental biology to disease modelling and tissue-remodelling studies [66], [67].

Long-term culture conditions can further improve metabolic and electrophysiological maturation [35], [68]. Extended culture has been associated with enhanced sarcomeric organization, improved calcium handling, increased connexin-43 expression, and partial metabolic transition toward oxidative phosphorylation [69], [70]. Similarly, epicardial–fibroblast interactions contribute to extracellular matrix remodelling and reparative signalling processes relevant to fibrosis and myocardial injury [71].

Recent bioengineering approaches, including perfusable culture systems and extracellular matrix optimization, have further enhanced the structural and functional maturation of cardiac organoid systems [70], [72]. However, these strategies should be distinguished from fully engineered cardiac tissues and organ-on-chip systems, in which tissue architecture and physiological conditions are externally imposed rather than intrinsically self-organized [73]. Despite substantial advances, current cardiac organoids remain developmentally immature and incompletely vascularized, limiting their ability to fully reproduce adult myocardial physiology [70], [72].

### 3.3 Engineered cardiac tissues and scaffold-based systems

Engineered cardiac tissues and scaffold-based systems represent externally guided cardiac platforms designed to support cellular organization, tissue maturation, and functional reproducibility. Unlike self-organizing cardiac organoids, these systems rely predominantly on biomaterial scaffolds and externally imposed structural cues rather than intrinsic developmental morphogenesis. As a result, scaffold-based platforms offer improved control over tissue architecture, mechanical properties, and experimental reproducibility, making them valuable tools for disease modelling, pharmacological testing, and regenerative applications [72], [74].

Natural biomaterials such as collagen, fibrin, and decellularized cardiac extracellular matrix (dECM) are widely used because they partially reproduce key biochemical and mechanical characteristics of the myocardial microenvironment [74], [75]. These matrices support cardiomyocyte adhesion, alignment, and contractile organization while facilitating extracellular matrix remodelling and tissue compaction during maturation [76], [77]. Decellularized ECM further preserves cardiac-specific structural proteins, anisotropic architecture, and vascular templates that enhance electrical coupling and sarcomeric organization [78].

Synthetic biomaterials, including polyethylene gly*co*l (PEG) and poly (lactic-co-glycolic acid) (PLGA), provide additional advantages through tuneable stiffness, degradation kinetics, and bioactive modification [79]. Hybrid scaffold systems combining natural and synthetic materials have demonstrated improved cardiomyocyte elongation, connexin-43 expression, and synchronized contractile behaviour, thereby supporting partial electrophysiological maturation of engineered cardiac tissues [80], [81]. Mechanical and structural properties of scaffolds also contribute significantly to tissue maturation. Parameters such as substrate elasticity, fiber alignment, and microvascular channel incorporation improve oxygen diffusion, endothelial integration, and long-term tissue viability [82], [83]. Advanced scaffold architectures integrating conductive biomaterials and aligned microstructures further enhance electromechanical coupling and coordinated contraction across engineered tissue [84], [85].

Despite these advances, scaffold-based cardiac systems remain engineering-directed constructs that do not fully reproduce the intrinsic developmental self-organization observed in cardiac organoids. Nevertheless, their scalability, reproducibility, and compatibility with biofabrication and regenerative strategies make them important complementary platforms for translational cardiovascular research [86].

### 3.4 3D bioprinted cardiac constructs

Three-dimensional bioprinting has emerged as an important engineering-based strategy for generating structurally organized cardiac tissue models with improved spatial control over cellular distribution, extracellular matrix (ECM) organization, and vascular architecture. Unlike self-organizing cardiac organoids, bioprinted cardiac constructs are externally designed systems in which tissue architecture is guided predominantly through biofabrication techniques rather than intrinsic developmental morphogenesis. Consequently, these platforms can provide greater reproducibility and geometric precision for cardiovascular tissue engineering and regenerative applications [87], [88], [89], [90].

Bioprinting approaches commonly utilize bioinks composed of cardiomyocytes, endothelial cells, fibroblasts, and ECM-derived hydrogels such as collagen, gelatin, fibrin, alginate, hyaluronic acid, and decellularized ECM [91], [92], [93]. These biomaterials support cellular adhesion, tissue organization, and partial electromechanical maturation while allowing programmable control over scaffold geometry and cellular positioning. Hybrid and chemically modified hydrogels, including GelMA-based systems, further improve structural stability and support tissue remodelling during maturation [93], [94], [95].

A major limitation of bioprinted cardiac systems remains the establishment of functional vascularization and long-term perfusion. To address this challenge, strategies such as sacrificial writing into functional tissue (SWIFT) and freeform reversible embedding of suspended hydrogels (FRESH) have been developed to generate perfusable microvascular-like networks within engineered tissues [96], [97], [98]. Incorporation of angiogenic growth factors, including vascular endothelial growth factor (VEGF) and basic fibroblast growth factor (bFGF), further supports endothelial organization and vascular remodelling. Advanced volumetric bioprinting and microgel-based bioinks have additionally improved structural stability and printing resolution, enabling fabrication of larger and more complex tissue architectures [99], [100], [101].

Bioprinted cardiac tissues also support partial electrophysiological and contractile maturation. Co-culture of human induced pluripotent stem cell-derived cardiomyocytes (hiPSC-CMs) with endothelial and stromal populations promotes cellular alignment, connexin-43 expression, and synchronized calcium handling within engineered constructs [94], [102], [103]. Scaffold-free approaches employing multicellular spheroids have similarly demonstrated the capacity to generate perfusable vascular-like networks and improve tissue integration following implantation [104], [105].

Despite substantial advances, current bioprinted cardiac constructs remain developmentally immature and dependent on externally imposed engineering conditions. Although these systems do not fully reproduce the intrinsic self-organization observed in cardiac organoids, their scalability, structural precision, and compatibility with patient-derived cells make them valuable complementary platforms for disease modelling, drug screening, and regenerative cardiovascular research.

### 3.5 Microfluidic cardiac organ-on-chip platforms

Microfluidic cardiac organ-on-chip platforms represent advanced engineering-based in vitro systems designed to recreate controlled biochemical, mechanical, and electrophysiological microenvironments relevant to human cardiac physiology [106], [107]. Unlike self-organizing cardiac organoids, organ-on-chip systems rely on externally imposed microfluidic architecture and biomechanical regulation to model cardiac tissue behaviour under defined experimental conditions [28], [108]. These platforms typically incorporate microchannels, flexible membranes, and perfusion systems that support regulated nutrient exchange, fluid flow, and mechanical stimulation [109], [110]. Most cardiac chips are fabricated using biocompatible materials such as polydimethylsiloxane (PDMS) and related polymers that permit integration of cardiomyocytes, fibroblasts, endothelial cells, and vascular-supporting populations within controlled microphysiological environments. Through combined biochemical and mechanical stimulation, these systems improve structural organization, calcium handling, electrophysiological synchronization, and partial maturation of human induced pluripotent stem cell-derived cardiomyocytes (hiPSC-CMs). Electrical stimulation and cyclic mechanical strain further enhance coordinated contraction and gap junction formation within engineered cardiac tissues [111], [112], [113].

In addition to maturation studies, cardiac organ-on-chip systems have become valuable tools for modelling cardiovascular diseases, including fibrosis, myocarditis, ischemic injury, vascular dysfunction, and drug-induced cardiotoxicity [6], [114]. Vascularized microfluidic platforms also permit investigation of endothelial signalling, inflammatory responses, thrombosis, and hemodynamic regulation under physiologically controlled conditions [115], [116]. Recent advances in integrated sensing technologies, muscle thin-film devices, and on-chip stem cell differentiation have further improved scalability and functional analysis for preclinical drug screening and disease modelling applications [117], [118]. Despite these advances, cardiac organ-on-chip systems remain dependent on externally engineered conditions and do not fully reproduce the intrinsic developmental morphogenesis observed in self-organizing cardiac organoids. Nevertheless, their controllability, reproducibility, and compatibility with high-throughput experimentation make them important complementary platforms for translational cardiovascular research.

## 4 Functional applications of human cardiac tissue models

**Fig. 2:**
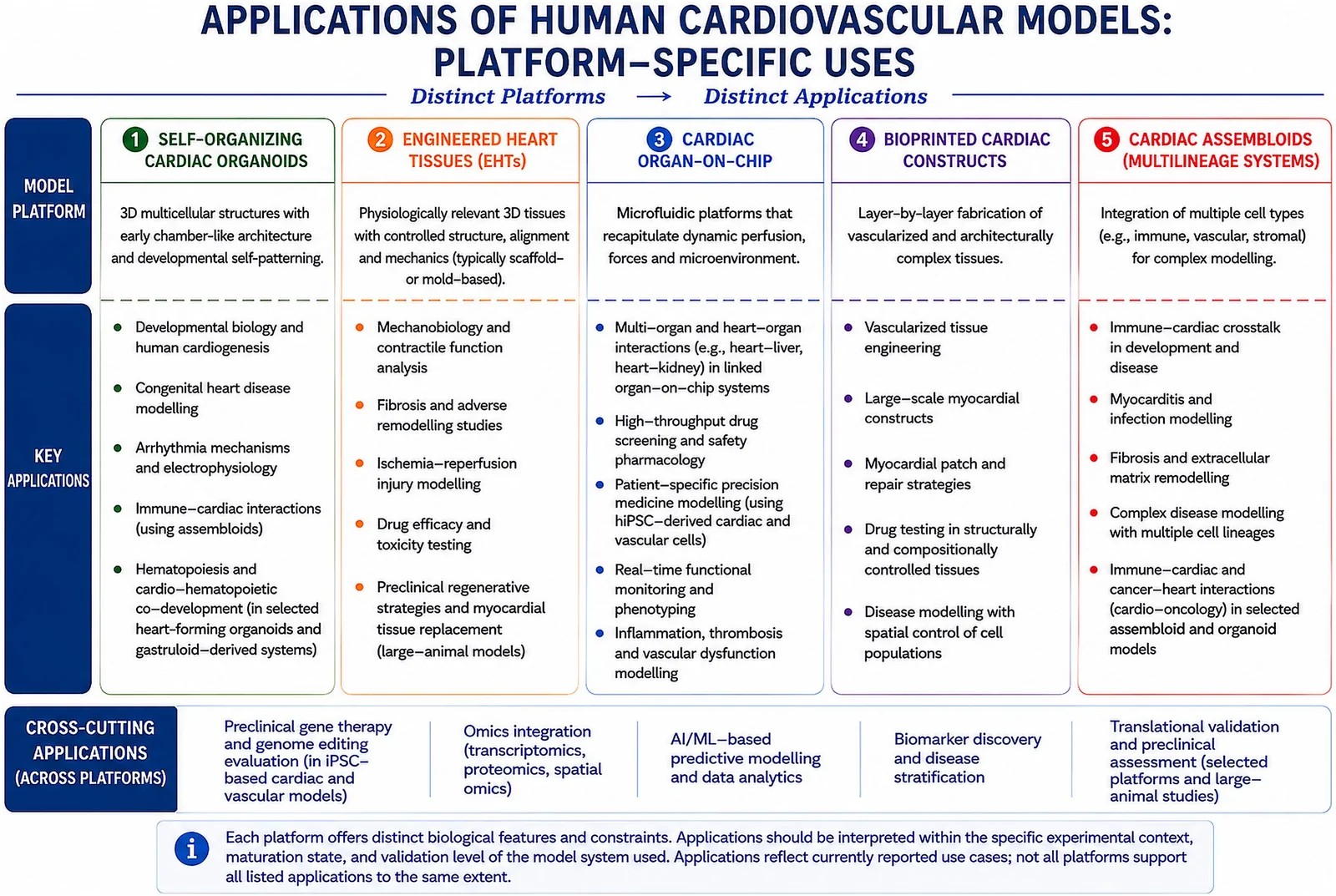
Platform-specific applications of human PSC-derived cardiac models. Overview of the principal applications of distinct human PSC-derived cardiac model platforms, including self-organizing cardiac organoids, engineered heart tissues, cardiac organ-on-chip systems, bioprinted cardiac constructs and cardiac assembloids. Representative applications include disease modelling, drug discovery and screening, cardiotoxicity assessment, regenerative medicine, gene therapy evaluation and bioengineering and tissue fabrication. Cross-platform applications, including omics, genome editing, artificial intelligence-based analysis and translational validation, are also indicated. The framework emphasizes that applications depend on the structural, cellular and functional properties of each platform and therefore should not be considered interchangeable.

Human PSC-derived cardiac organoids, engineered heart tissues, and related microphysiological platforms provide experimentally relevant systems for studying cardiac development, disease progression, pharmacological responses, and regenerative mechanisms within multicellular cardiac microenvironments. By integrating structural, electrophysiological, and molecular features, these platforms have expanded applications in disease modelling, drug screening, cardiotoxicity assessment, gene therapy evaluation, and regenerative cardiovascular research (Fig. 2). However, despite significant advances, current models remain limited by developmental immaturity, incomplete vascularization, batch variability, and restricted long-term physiological stability.

### 4.1 Cardiovascular disease modelling

Human cardiac organoids (hCOs) have emerged as valuable platforms for modelling selected aspects of cardiovascular disease by reproducing relevant developmental and pathological features of the human heart. These three-dimensional multicellular systems can incorporate cardiomyocytes, endothelial, fibroblast, and stromal populations, enabling investigation of cell–cell interactions and tissue-level processes that are difficult to reproduce in conventional two-dimensional cultures. Temporal modulation of developmental signalling pathways, particularly WNT signalling, enables the generation of self-organizing cardiac organoids and cardioids. These models reproduce key features of myocardial infarction, including sarcomeric disruption, inflammatory activation, calcium dysregulation, and metabolic impairment [119], [120], [121], [122].

Several cardiovascular disorders have been investigated using 3D cardiac systems. Ischemic injury has been modeled using distinct platforms, including human cardiac organoids exposed to diffusion limitations and adrenergic stress [123] and microphysiological border-zone-on-a-chip systems incorporating spatial oxygen gradients [124] reproduce key aspects of myocardial infarction, including sarcomeric disruption, inflammatory activation, calcium dysregulation, and metabolic impairment.

Similarly, fibrotic remodelling induced by transforming growth factor-β (TGF-β) promotes extracellular matrix accumulation and tissue stiffening, enabling investigation of fibrosis-associated cardiac dysfunction. Complementing these inducible chemical models, engineered cardiac tissues and 3D cardiac microtissues carrying desmoplakin (DSP) mutations have additionally demonstrated impaired contractility, desmosomal instability, and fibrotic remodelling characteristic of arrhythmogenic cardiomyopathy [125], [126]. Self-organizing human heart organoids have also emerged as valuable platforms for investigating diabetes-associated developmental heart disease. Human heart organoids cultured under pregestational diabetes (PGD)-like conditions reproduce key features of diabetic embryonic cardiomyopathy, including cardiomyocyte (CM) hypertrophy, oxidative and mitochondrial stress, impaired contractile activity, altered cardiac developmental gene expression, and disrupted microvascular organization. Single-cell RNA sequencing further revealed altered cardiac cell composition, reduced CM representation, expansion of epicardial populations, and disrupted cell–cell signalling. Mechanistically, endoplasmic reticulum (ER) stress activated inositol-requiring enzyme 1 (IRE1) and regulated IRE1-dependent decay, leading to degradation of fatty acid desaturase 2 and impaired synthesis of omega-3 polyunsaturated fatty acids. Pharmacological and metabolic interventions targeting this pathway, including KIRA8, tauroursodeoxycholic acid, tetrahydrobiopterin, and omega-3 fatty acids, alleviated several pathological phenotypes. These findings highlight the utility of self-organizing human heart organoids for elucidating mechanisms of pregestational diabetes-associated congenital heart disease and evaluating potential therapeutic strategies [127].

Electrophysiological disorders have also been modeled using patient-derived induced pluripotent stem cell-derived cardiomyocytes (iPSC-CMs) and 3D engineered cardiac tissues. Systems carrying CASQ2 or RYR2 mutations exhibit abnormal calcium handling, prolonged field potential duration, and conduction abnormalities associated with inherited arrhythmias when evaluated via microelectrode arrays (MEAs) and biomechanical EHT platforms [126], [128], [129]. In parallel, engineered cardiac tissues subjected to chronic β-adrenergic stimulation reproduce key features of pathological hypertrophic remodelling including impaired contractile function, activation of fetal cardiac gene programs (e.g., *NPPA* and *NPPB*), and structural remodelling, thereby providing a physiologically relevant model of heart failure progression [130].

In preclinical models of myocardial infarction, engineered cardiac tissue patches incorporating extracellular matrix scaffolds and vascular-supporting cell populations have demonstrated enhanced cell engraftment, improved neovascularization, and partial functional recover [131]. Complementing these tissue-engineering graft strategies, targeted hormonal supplementation has been shown to induce functional T-tubule development and structural maturation in hiPSC-derived cardiomyocytes [132]. Engineered heart tissues and bioengineered cardiac constructs have provided important platforms for regenerative and functional studies. Scaffold- or matrix-based cardiac constructs incorporating extracellular matrix components and vascular-supporting cell populations have been investigated in preclinical models of myocardial injury, with approaches aimed at improving cell retention, vascularization, tissue integration, and functional recovery. In parallel, maturation strategies for hiPSC-derived cardiomyocytes and engineered cardiac tissues—including metabolic conditioning, hormonal cues such as thyroid hormone, niche-cell or vascular co-culture, and electrical or mechanical stimulation—have been used to improve structural organization, electrophysiological properties, contractile performance, and physiological maturity [132], [133], [134], [135].

In addition, recent advances have expanded cardiovascular organoid platforms into increasingly complex multicellular systems. Blood-generating heart-forming organoids (BG-HFOs) provide a unique developmental platform for modelling coordinated embryonic heart and hematopoietic co-development, thereby enabling investigation of cardio-hematopoietic interactions during early human development [31]. In parallel, immunocompetent cardiac assembloids, neural crest-integrated cardiac models, and vascularized multi-tissue platforms have further broadened the utility of cardiovascular organoid systems for investigating immune–cardiac interactions, cardiac development, cell–cell communication, and multicellular mechanisms underlying cardiovascular disease progression and therapeutic responses [32], [136], [137].

Despite these advances, current cardiac organoid systems do not fully reproduce adult myocardial physiology or long-term systemic interactions. Therefore, they should presently be considered complementary preclinical platforms rather than complete replacements for established in vivo cardiovascular models.

### 4.2 Drug screening and toxicological evaluation

Human vascularized and chambered cardiac organoids, together with hiPSC-derived cardiac organoid-based functional screening platforms, have become increasingly valuable systems for evaluating pharmacological efficacy, cardiotoxicity, and disease-associated drug responses under multicellular, human-relevant conditions. Compared with conventional two-dimensional cultures, these platforms permit integrated assessment of contractility, electrophysiological behaviour, metabolic activity, and tissue-level toxicity within structurally organized cardiac microenvironments [138], [139].

Several studies have demonstrated the utility of human cardiac organoids and cardiac spheroids for modelling infection-associated and developmental cardiotoxicity. In SARS-CoV-2–infected human cardiac organoids, bromodomain-containing protein 4 was identified as an important regulator of inflammatory and viral-response pathways, while BET inhibition reduced cardiomyocyte injury and viral-associated dysfunction [140], [141]. Developmentally guided human cardiac organoids have similarly reproduced structural abnormalities associated with teratogenic drug exposure, including ondansetron-associated ventricular developmental defects [58]. In hypoxia-exposed hiPSC-cardiomyocytes and cardiac spheroids, doxorubicin exposure further exacerbated hypoxia-induced cardiotoxicity and metabolic stress, supporting the relevance of these systems for studying combined pathological and pharmacological injury; related work has also used patient-specific iPSC-derived heart organoids to model doxorubicin-induced cardiotoxicity [142], [143].

Human cardiac organoids, vascularized cardiac organoids, and cardiac organoid-on-chip systems have additionally been applied to investigate metabolic, inflammatory, and vascular responses to pharmacological agents. hiPSC-derived cardiac organoids cultured under high-glucose and high-lipid conditions have recapitulated key features of diabetic cardiomyopathy, including cardiomyocyte hypertrophy, mitochondrial dysfunction, and fibrotic remodelling. Vascularized cardiac organoids incorporating endothelial and mural support cells, as well as vascularized cardiac spheroid-on-chip platforms, have further enabled investigation of endothelial dysfunction, angiogenic remodelling, and drug-induced vascular responses, while microfluidic perfusion systems and long-term organoid culture platforms have improved chronic toxicity assessment and longitudinal drug exposure studies [24], [144], [145], [146].

Recent high-throughput approaches integrating automated 3D imaging, optical contractility analysis, and multi-electrode array (MEA) electrophysiology have improved the scalability of human PSC-derived cardiac models for drug screening [138], [147]. Multi-omics profiling has further identified molecular signatures associated with oxidative stress, mitochondrial dysfunction, extracellular matrix remodelling, and calcium-handling abnormalities following pharmacological exposure [148], [149], [150]. These integrated approaches support mechanistic toxicology and preclinical therapeutic evaluation.

Future refinement of cardiac organoids and engineered cardiac platforms through electrical stimulation, immune integration, multi-organ coupling, and AI-assisted analytics may further improve disease-specific drug testing and personalized pharmacological assessment [146], [151], [152], [153]. Nevertheless, variability in maturation status, tissue reproducibility, and long-term functional stability continues to limit direct clinical and regulatory translation.

### 4.3 Gene therapy applications in human cardiac tissue models

Patient-specific iPSC-derived cardiac models generated from induced pluripotent stem cells have emerged as valuable platforms for evaluating gene therapy and precision medicine strategies in inherited cardiac disorders. Their three-dimensional architecture, together with preservation of patient-specific genetic backgrounds, enables assessment of disease phenotypes, genome-editing efficiency, and functional recovery following therapeutic intervention. Restoration of wild-type TAZ expression in patient-derived iPSC-cardiomyocytes and engineered “heart-on-chip” tissues representing Barth syndrome improved sarcomeric organization and contractile function, highlighting the potential of these human iPSC–based platforms for evaluating gene replacement strategies. In complementary murine studies, AAV-mediated TAZ gene replacement in TAZ-knockout mice prevented and reversed heart failure, supporting the therapeutic concept in an in vivo model [154], [155]. Likewise, CRISPR/Cas9-mediated in vivo editing of pathogenic RYR2 variants in a mouse model of catecholaminergic polymorphic ventricular tachycardia normalized calcium handling and markedly reduced arrhythmogenic activity, illustrating the potential of somatic genome editing in cardiac disease. Reviews of CRISPR-based approaches further summarize how such in vivo and iPSC-based cardiac models are being used to model and correct cardiovascular mutations [156], [157].

Beyond these examples, complementary gene-editing strategies, including prime editing, antisense oligonucleotide therapy, and AAV-mediated gene delivery have been investigated in patient-specific iPSC-derived cardiomyocytes and engineered cardiac models for the treatment of inherited cardiomyopathies and channelopathies [155], [158], [159], [160], [161]. These human cardiac models preserve disease-associated genetic backgrounds, enabling quantitative assessment of genome-editing efficiency, electrophysiological recovery, contractile function, and potential cellular responses following therapeutic intervention. Studies involving Duchenne muscular dystrophy-associated cardiomyopathy in patient-derived 3D bioprinted cardiac organoid constructs, Long QT syndrome in patient-specific iPSC-derived cardiomyocytes, and catecholaminergic polymorphic ventricular tachycardia in iPSC-based and in vivo models have demonstrated partial restoration of dystrophin expression, normalization of calcium handling, and improved action potential stability after targeted genetic correction [128], [161], [162].

Recent advances in vascularized cardiac organoids, microphysiological platforms, and multi-omics analyses have enhanced the utility of patient-specific cardiac models for evaluating gene-editing efficacy, tissue maturation, and therapeutic responses [22], [24], [123]. Nevertheless, challenges related to editing efficiency, off-target effects, delivery specificity, incomplete tissue maturation, and long-term safety remain to be addressed. Therefore, patient-specific iPSC-derived cardiac models, including organoids and engineered microphysiological platforms, currently serve as complementary preclinical systems for optimizing gene therapy strategies rather than substitutes for clinical validation [11], [16], [163].

### 4.4 .hPSC-derived cardiac platforms for myocardial repair

Human pluripotent stem cell (hPSC)-derived engineered heart tissues (EHTs), cardiac muscle patches, and cardiac organoids have shown considerable potential as bioengineered grafts for myocardial repair following ischemic injury [57], [131], [164]. Compared with conventional two-dimensional cultures, vascularized three-dimensional engineered cardiac constructs and cardiac organoids exhibit improved cell survival, structural maturation, electromechanical integration, and angiogenic potential after transplantation. In preclinical myocardial infarction models, hiPSC-derived cardiac muscle patches and human cardiac organoids have been associated with reduced scar formation, enhanced neovascularization, and improved ventricular function through both direct tissue replacement and paracrine signalling [131], [135]. Recent studies have further demonstrated that hypoimmunogenic hPSC-derived cardiac organoids and HLA-engineered hPSC-cardiomyocytes prolong graft survival and reduce immune rejection in preclinical models. Although early preclinical and translational studies using clinical-grade hiPSC-derived cardiomyocyte patches have demonstrated encouraging safety and feasibility, challenges related to graft maturation, immune compatibility, arrhythmogenicity, long-term integration, and scalable manufacturing remain to be overcome before widespread clinical translation [165].

## 5 Ethical and regulatory considerations in cardiovascular organoid research

**Fig. 3:**
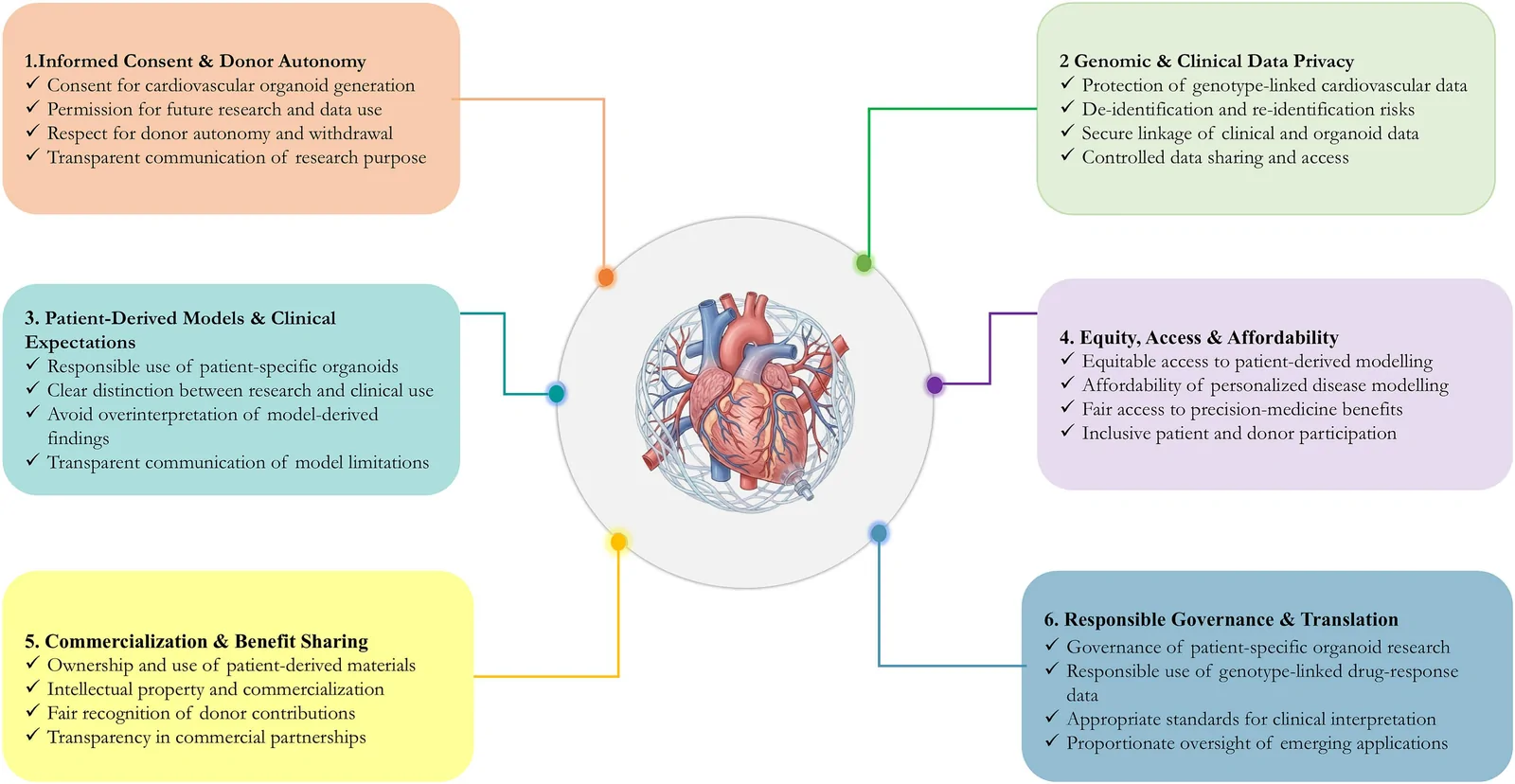
Ethical, governance and translational considerations for human cardiovascular organoid research. Framework highlighting key ethical and governance considerations associated with human cardiovascular organoid research, particularly patient- and donor-derived models. The major domains include informed consent and donor autonomy, genomic and clinical data privacy, patient-derived models and clinical expectations, equity and access, commercialization and benefit sharing, and responsible governance and translation. The framework emphasizes responsible use of patient-derived materials and genotype-linked information, appropriate communication of model limitations, equitable access to emerging applications and proportionate governance of translational research.

The rapid progression of cardiovascular organoid research from developmental modelling toward regenerative and translational applications has introduced important ethical and regulatory challenges that extend beyond conventional stem cell research frameworks. Unlike traditional in vitro cardiac models, cardiovascular organoids increasingly incorporate patient-derived multicellular architecture and clinically relevant genomic information, thereby raising important ethical and regulatory questions related to donor autonomy, genomic privacy, commercialization, and equitable clinical access. Consequently, ethical oversight should extend beyond donor consent and data governance to include governance of patient-derived biological materials, responsible commercialization, equitable access, and regulatory frameworks for safe clinical translation (Fig. 3).

### 5.1 Ethical standards and data governance

Cardiovascular organoids are commonly derived from patient-specific induced pluripotent stem cells (iPSCs), which retain the donor's genomic identity and disease-associated characteristics. This raises important ethical questions regarding informed consent, long-term data use, genomic privacy, and ownership of derived biological materials. Because cardiovascular organoid research frequently involves biobanking, repeated experimental use, and integration of genomic and electrophysiological datasets, conventional one-time consent models may be insufficient for long-term translational studies. Dynamic or tiered consent approaches have therefore been proposed to allow donors greater control over future use of their biological samples and associated clinical data [166], [167].

Protection of donor privacy represents another major ethical consideration. Cardiovascular organoids may contain identifiable genomic and phenotypic information linked to inherited cardiomyopathies, arrhythmia susceptibility, drug responses, and other patient-specific traits. Integration of artificial intelligence (AI)-assisted phenotyping, automated electrophysiological analysis, and multi-omics profiling further increases the complexity of data governance. Consequently, secure data management systems, controlled-access repositories, and transparent policies regarding data sharing and AI-assisted analysis are required to minimize risks of unauthorized re-identification and misuse of sensitive genomic information [167], [168], [169].

International regulatory frameworks, including the 2021 ISSCR Guidelines for Stem Cell Research and Clinical Translation and the Indian Council of Medical Research (ICMR) National Guidelines for Stem Cell Research, provide comprehensive ethical and regulatory guidance for stem cell procurement and derivation, organoid research, biobanking, institutional oversight, and responsible clinical translation [170], [171], [172]. These frameworks emphasize scientific validity, institutional review, informed consent, and transparency in the handling of patient-derived materials. As cardiovascular organoid systems become increasingly integrated into translational and preclinical pipelines, harmonization of ethical reporting standards and quality-control practices will become increasingly important for reproducibility and regulatory consistency across institutions and jurisdictions.

### 5.2 Translational ethics and commercialization

The clinical translation of human cardiovascular organoids, engineered cardiac tissues, and microphysiological platforms from experimental disease models to regenerative therapies and precision medicine introduces several ethical and regulatory challenges that extend beyond laboratory research [167], [173], [174]. Unlike conventional in vitro systems, patient-derived cardiovascular organoid and engineered tissue models may ultimately inform individualized therapeutic decisions or serve as components of regenerative interventions [72], [123]. Consequently, ethical oversight should ensure that clinical application is supported by robust preclinical evidence demonstrating safety, efficacy, and functional reliability before first-in-human studies. Regulatory authorities should therefore require rigorous preclinical safety evaluation and standardized clinical oversight before first-in-human application to ensure responsible clinical translation [174], [175].

Commercialization of patient-specific cardiovascular organoid technologies presents additional ethical challenges related to ownership of patient-derived biological materials, intellectual property rights, benefit sharing, affordability, and the long-term rights of donors over organoids generated from their tissues [167]. Because organoids generated from patient-specific induced pluripotent stem cells possess substantial scientific and commercial value, transparent policies are required to define ownership, licensing, and the responsible use of these biological resources. Ethical commercialization frameworks should ensure that patients are appropriately informed regarding future clinical and commercial applications of their donated tissues, while promoting transparency, benefit sharing where appropriate, and public trust in translational research [167], [168], [173], [176], [177].

Cardiovascular organoids and iPSC-derived cardiac platforms are increasingly being explored to predict genotype-linked drug responses and support precision cardiovascular medicine. Although these advances offer significant clinical potential, they also raise ethical concerns regarding protection of genotype-associated health information, secondary use of patient-derived datasets, and potential misuse of predictive clinical data [178], [179]. Appropriate governance mechanisms, including secure data management, controlled-access repositories, transparent data-sharing policies, and safeguards against misuse of genotype-linked clinical information, will therefore be essential to protect patient privacy while enabling scientific innovation [180], [181].

Equitable access represents an important ethical consideration in the translation of cardiovascular organoid technologies. The generation and clinical-grade manufacture of patient-specific cardiovascular organoids or iPSC-derived cardiac products require specialized infrastructure, skilled personnel, prolonged culture and differentiation procedures, quality-control testing, and costly reagents. These technical and financial requirements may restrict implementation to well-resourced institutions and healthcare systems, thereby worsening disparities in access to personalized cardiovascular diagnostics and therapies. Accordingly, regulators, healthcare providers, payers, researchers, and industry should collaborate to develop evidence-based reimbursement models, fair commercialization and intellectual-property policies, standardized manufacturing and quality-control procedures, and implementation strategies suited to resource-constrained settings. Such measures should promote affordability and equitable access without compromising product quality, efficacy, or patient safety [182], [183], [184], [185].

As cardiovascular organoid and iPSC-derived cardiac technologies progress toward translational and, potentially, clinical applications, ethical governance must evolve alongside scientific development [167], [186]. Future governance frameworks should balance scientific innovation with respect for patient autonomy, protection of genomic privacy, responsible commercialization, equitable access, fair benefit sharing, and rigorous assessment of clinical validity, safety, and efficacy [181], [187], [188]. Such frameworks should also address informed consent for secondary use of donor-derived materials, traceability and re-identification risks, manufacturing consistency, long-term monitoring, and accountability among researchers, healthcare providers, regulators, and industry. An integrated ethical and regulatory approach will therefore be essential for ensuring the safe, transparent, equitable, and socially responsible translation of cardiovascular organoid technologies into biomedical research and clinical practice [167], [178].

## 6 Future directions

Future progress in cardiovascular organoid research will depend on improving physiological maturation while preserving the developmental self-organization that distinguishes organoids from engineered cardiac tissues. Although current-generation human cardiac organoids reproduce key aspects of cardiac morphogenesis and disease, most models retain immature electrophysiological, metabolic, and structural characteristics, limited vascular perfusion, and considerable batch-to-batch variability. Future efforts should therefore prioritize optimized differentiation protocols, electrical and mechanical conditioning, metabolic maturation, and chemically defined culture systems to generate more reproducible and adult-like cardiac tissues [189], [190], [191].

A major future direction is the development of increasingly complex, multi-lineage cardiovascular organoids that more faithfully recapitulate the native cardiac microenvironment. While conventional human cardiac organoids reproduce fundamental aspects of myocardial development, they generally lack functional immune, hematopoietic, and neuroectodermal cell populations [144]. Recent advances have addressed these limitations through developmentally guided differentiation strategies that increase cellular diversity and physiological relevance. Immunocompetent heart assembloids have enabled investigation of immune–cardiac interactions during tissue injury and repair [32], whereas neural crest-containing cardiac assembloids have expanded opportunities to investigate congenital heart disease, cardiac innervation, and neurocardiac signalling. Similarly, cardio-hematopoietic organoids containing CD45^+^ hematopoietic cells have provided new opportunities to study hematopoietic specification within the developing cardiac niche. More recently, vascularized heart–liver organoids generated using micropatterned gastruloids and vascular-inducing morphogens have demonstrated the feasibility of modelling tissue perfusion and inter-organ communication within integrated developmental systems [31], [144], [151]. Collectively, these next-generation platforms represent an important transition from relatively simple cardiac organoids toward developmentally integrated, multi-lineage cardiovascular models with greater physiological fidelity and translational relevance.

Future translational progress will also depend on integrating cardiovascular organoids with complementary bioengineering technologies while preserving their developmental identity. Microfluidic organ-on-chip systems, perfusion bioreactors, and advanced three-dimensional bioprinting provide controlled mechanical stimulation, continuous nutrient perfusion, and precise spatial organization that collectively enhance tissue maturation, functional stability, and reproducibility [35], [192], [193], [194], [195]. In parallel, structure–function co-design strategies integrating developmental biology with biomaterial engineering are expected to optimize tissue architecture, vascular organization, and electromechanical coupling, thereby improving the physiological fidelity and predictive value of cardiovascular organoid models for disease modelling and therapeutic evaluation [22], [196].

Standardization will remain equally important for successful clinical translation. Harmonized differentiation protocols, defined quality-control criteria, standardized electrophysiological and structural benchmarks, and good manufacturing practice (GMP)-compatible production pipelines are needed to improve reproducibility across laboratories. In parallel, integration of multi-omics profiling with artificial intelligence-assisted phenotyping and computational modelling is expected to enable comprehensive characterization of organoid maturation, cellular heterogeneity, and patient-specific therapeutic responses while supporting the development of digital-twin frameworks for precision cardiovascular medicine.

Despite these advances, significant challenges remain, including incomplete tissue maturation, durable vascular integration, immune compatibility, graft-host electrical coupling, manufacturing scalability, regulatory standardization, and long-term safety. Consequently, cardiovascular organoids should presently be regarded as complementary human-specific preclinical platforms that bridge conventional cell culture and animal models rather than replace them. Continued collaboration among developmental biologists, stem cell researchers, bioengineers, computational scientists, clinicians, and regulatory agencies will be essential for establishing robust, standardized, and clinically translatable cardiovascular organoid systems.

## 7 Conclusion

Human cardiovascular organoids and engineered cardiac tissue systems have emerged as valuable experimental platforms for investigating cardiac development, disease mechanisms, pharmacological responses, and regenerative strategies within multicellular human-relevant environments. Advances in stem cell biology, tissue engineering, microfluidics, and biofabrication have substantially improved the structural and functional complexity of these systems, expanding their applications in disease modelling, cardiotoxicity assessment, precision medicine, gene therapy evaluation, and regenerative cardiovascular research.

The development of self-organizing cardiac organoids, engineered cardiac tissues, bioprinted constructs, and organ-on-chip platforms has further enhanced the ability to model human cardiac physiology under experimentally controlled conditions. These technologies have provided important insights into inherited cardiomyopathies, arrhythmogenic disorders, ischemic injury, fibrotic remodelling, and therapeutic responses that are not fully reproduced by conventional two-dimensional culture systems. In parallel, integration of vascular, stromal, immune, and computational components has improved the translational relevance of emerging cardiovascular organoid platforms.

Despite these advances, cardiovascular organoids remain limited by developmental immaturity, incomplete vascularization, reproducibility issues, and challenges in long-term stability, immune compatibility, electromechanical integration, arrhythmogenic risk, and manufacturing standardization. Consequently, they currently serve as complementary preclinical models rather than direct substitutes for native human cardiac tissue or in vivo models. Continued progress in cardiovascular organoid research will depend on interdisciplinary collaboration across stem cell biology, bioengineering, computational modelling, translational medicine, and regulatory science. Further optimization of tissue maturation, vascular integration, electrophysiological stability, scalable manufacturing, and ethical governance will be essential for improving reproducibility and translational applicability. With careful scientific validation and responsible clinical development, cardiovascular organoids may contribute substantially to future advances in precision cardiovascular research, therapeutic screening, and regenerative medicine.

## CRediT authorship contribution statement

**Koushik Sen:** Writing – original draft, Methodology, Investigation. **Suravi Majumder:** Writing – review & editing, Writing – original draft, Visualization, Validation, Supervision, Resources, Methodology, Investigation, Funding acquisition, Formal analysis, Data curation, Conceptualization. **Dragana Stanisic:** Writing – original draft. **Avisek Majumder:** Writing – original draft.

## Ethical statement

### Informed consent

The generation of human cardiovascular organoids (hCOs) discussed in this review relies on human pluripotent stem cells (hPSCs) and induced pluripotent stem cells (iPSCs). The authors emphasize that the primary research cited in this manuscript was conducted under frameworks requiring informed consent, donor autonomy, and ongoing participation through dynamic consent systems. Informed consent for this specific review article was not applicable as no new human data were created.

### Human and animal rights

As this is a review article, it does not contain any original studies with human participants or animals performed by any of the authors. The review synthesizes findings from previously published literature, which were conducted in accordance with the ethical standards of the institutional and/or national research committees and with the 1964 Helsinki Declaration and its later amendments.

### Data privacy and governance

The authors acknowledge the critical importance of protecting sensitive genomic and personal data generated in organoid research. This review highlights the necessity of secure digital repositories and international cooperation to align with regulations such as the General Data Protection Regulation (GDPR).

### Use of emerging technologies

The authors declare that the discussion regarding Artificial Intelligence (AI) and CRISPR-based genome editing in this review is framed within responsible innovation guidelines. Efforts were made to address potential algorithmic bias and the ethical implications of complex human tissue models.

## Funding

This research received no specific grant from any funding agency in the public, commercial, or not-for-profit sectors.

## Declaration of competing interest

The authors declare that they have no known competing financial interests or personal relationships that could have appeared to influence the work reported in this paper.
